# A Cross-Sectional Survey on the Malaria Control and Prevention Knowledge, Attitudes, and Practices of Caregivers of Children Under-5 in the Western Area of Sierra Leone

**DOI:** 10.3390/tropicalmed7070120

**Published:** 2022-06-28

**Authors:** Joan Mabinty Koroma, Yuji Wang, Xiang Guo, Xiaoqing Zhang, Jone Jama Kpanda Ngobeh, Ahmed Mohamed Elamin Ali Gabir, Ziyao Li, Li Li, Rangke Wu, Xiaohong Zhou

**Affiliations:** 1 Department of Pathogen Biology, Institute of Tropical Medicine, School of Public Health, Southern Medical University, Guangzhou 510515, China; jmk2305@cumc.columbia.edu (J.M.K.); sureji@i.smu.edu.cn (Y.W.); guo601765739@i.smu.edu.cn (X.G.); zxq1520049434@i.smu.edu.cn (X.Z.); jone.ngobeh@gmail.com (J.J.K.N.); ahmedmgabir@gmail.com (A.M.E.A.G.); leezyao@163.com (Z.L.); 2 State Key Laboratory of Organ Failure Research, Department of Biostatistics, Guangdong Provincial Key Laboratory of Tropical Disease Research, School of Public Health, Southern Medical University, Guangzhou 510515, China; lylygdsg@smu.edu.cn; 3 The School of Foreign Studies, Southern Medical University, Guangzhou 510515, China; fanglan@smu.edu.cn

**Keywords:** malaria, Sierra Leone, knowledge, attitudes, and practices (KAPs), caregivers, children under-5, community-based intervention

## Abstract

(1) Background: Children under 5 years of age are the most vulnerable to malaria infection, and they suffer serious complications. Sierra Leone is one of the countries with the highest malaria burden in the world. This study aimed to assess the knowledge, attitudes, and practices (KAPs) toward malaria control and prevention among caregivers of children under 5 in the Western Area of Sierra Leone. (2) Methods: A cross-sectional survey was conducted among caregivers of children under-5 visiting the out-patient department of six selected hospitals/community health centers. Data were collected via questionnaire interviews with 350 caregivers. (3) Results: A total of 97.1% of the respondents were women and the majority of them were young mothers; 46.3% of respondents were unemployed; and 27.1% received no education. Only 1.4% accessed malaria related information from the internet/social media. This KAPs survey indicated that a misconception of the cause, transmission, and clinical symptoms of malaria; unawareness of its lethality and its severity; and inappropriate prevention and treatment behaviors, such as self-medicating, were still in existence among some caregivers. However, a positive correlation in knowledge–attitudes (r_s_ = 0.13, *p* < 0.05) and in attitudes–practices (r_s_ = 0.45, *p* < 0.001) was revealed. The caregivers, being mothers and having at least a secondary education, demonstrated positive attitudes and practices. Meanwhile, more urban caregivers (79.8%) followed a complete malaria treatment course of artemisinin-based combination therapies than the rural (63.3%), but in view of insecticide treated net use, more rural caregivers presented positive attitudes (85.3%) and practices (70.1%) than the urban (69.9%, 52.0%). (4) Conclusions: For better protection of children under-5 against lethal malaria, it is essential to provide better guidance at the community level for their caregivers, especially young mothers, in order to reduce some misconceptions and inappropriate behaviors. An increase in education and employment opportunities for women, establishment of an accessible community-based malaria counselling service, and construction of an effective communication channel are also needed.

## 1. Introduction

Malaria remains globally endemic in 85 countries, and it is mostly concentrated in the underdeveloped tropical and subtropical regions even though its occurrences vary with geographic–climatic environmental conditions and cultural–hygienic social factors [1,2]. Compared with 2019, the estimated malaria cases and deaths increased to 241 million and 627,000, respectively, in 2020, and approximately 95% of cases and 96% of deaths occurred in the WHO African Region, in which Sierra Leone had a heavy malaria burden, with an estimated 2,617,968 cases and 8054 deaths [1]. An estimated 77% of all malaria deaths occurred in children under 5 years of age, mostly in Sub-Saharan Africa. The entire population in Sierra Leone is at risk of malaria, but children under-5 and pregnant women are the most vulnerable groups. Etiologically, malaria is mainly transmitted through the infected *Anopheles* mosquitoes. *Plasmodium falciparum* is responsible for over 90% of malaria cases and all the severe types of the disease in Sierra Leone [1,3].

In the postwar and the post-Ebola periods, malaria control efforts in Sierra Leone are hampered by inadequate capacity due to the country’s weak health care system, the unhealthy hygiene practices of people, and other poverty-related social and environmental conditions. In remote rural areas and in swampy regions, health facilities are less accessible to inhabitants, with consequent devastations to their health due to malaria [4]. Although malaria is curable using artemisinin-based combination therapies (ACTs), its prevention and its control still poses a challenge to public health systems at the global, national, subnational, and community levels, and it poses a huge health burden on both the government and on individuals, subsequently hindering socioeconomic development and even leading to deeper poverty [5]. Furthermore, severe malaria impairs children’s learning and cognitive abilities by as much as 60%, consequently affecting the performance of Sierra Leone’s primary and secondary education programs [6].

Over the past years, Sierra Leone has made some progress in controlling malaria. Such progress has included the development of a National Malaria Control Programme (NMCP) Strategic Plan 2016–2020 to ensure that the implemented program is evidence-based [6]. Based on the Global Malaria Programme (GMP) [7], the WHO Malaria Report 2021 [1] and the Global Technical Strategy for Malaria 2016–2030 (GTS) [8], community empowerment, capacity-building, and supportive supervision from a strong health workforce are considered important to ensure the effectivity of the health system at national, district, and community levels to reduce and to eliminate malaria mortality and morbidity.

However, there still exists such incomplete knowledge and misconceptions as well as misconduct regarding malaria, such as increased exposure to the disease, inappropriate implementation of interventions, and inadequate recognition of the factors contributing to the occurrence and the development of malaria, which probably hinder the correct practices against it [9]. Research [10,11,12,13] concerning individual knowledge, attitudes, and practices (KAPs) has shown that the factors, including education levels in some instances, are related to malaria prevention and control behaviors. At the community level, antimalaria behaviors are affected by KAPs levels, which is even more crucial [14]. 

Primary caregivers play key roles in the health care of children under-5, being responsible for a majority of the everyday practices and decisions that determine childrens’ health status. A caregiver’s KAPs level toward protecting their children from malaria is essential for seeking appropriate medical care in case of the onset of the disease and administering recommended treatments [15]. Thus, in the present study, an insight into the KAPs of caregivers toward malaria may contribute to evidence-based malaria prevention and control the well-informed design of community interventions in order to reduce the prevalence of malaria in children under-5.

## 2. Materials and Methods

### 2.1. Setting

This study was carried out in the Western Area, one of four principal divisions of Sierra Leone. The Western Area comprises the oldest city and the national capital, Freetown, and its surrounding towns and countryside, and it covers an area of 557 km^2^ with a total population of approximately 1.5 million people (2015 National census). The Western Area is divided into two districts: Western Area Rural (WAR) and Western Area Urban (WAU).

**Figure 1:**
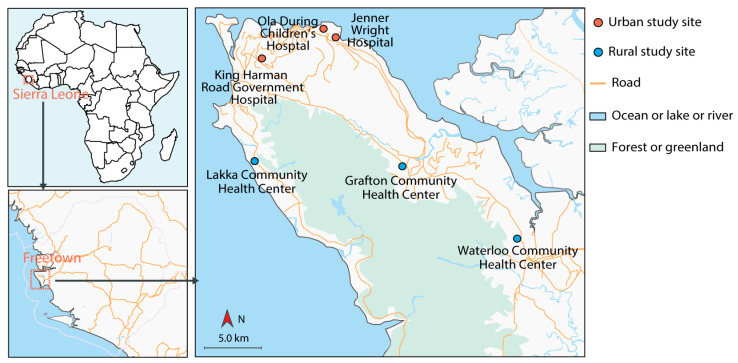
Geographic location of the study sites. Health care facilities in WAU included: Ola During Children’s Hospital (ODCH) (8°29′25″ E, 13°13′7″ S), King Harmann Road Government Hospital (8°28′27″ E, 13°14′52″ S), and Jenner Wright Hospital (8°29′7″ E, 13°12′43″ S). Health care facilities in WAR included: Waterloo Community Health Center (8°20′08″ E, 13°4′24″ S), Grafton Community Health Center (8°23′34″ E, 13°9′23″ S), and Lakka Community Health Center (8°23′49″ E, 13°15′50″ S).

We conducted pilot investigations in WAR and WAU for selection of the suitable and the representative survey sites. Consequently, government-owned hospitals or health centers have been selected as they are strategically located within easy reach of the majority of the communities, and they provide free health services for children under-5, and they almost always have a large number of caregivers for children under-5 accessing their healthcare services. Therefore, this study was carried out in six selected health care facilities in two districts, WAU and WAR (Figure 1), three from WAU: Ola During Children’s Hospital (ODCH), King Harmann Road Government Hospital, and Jenner Wright Hospital; and the other three were from WAR: Waterloo Community Health Center, Grafton Community Health Center, and Lakka Community Health Center.

### 2.2. Study Population and Eligibility Criteria

Caregivers with one or more child(ren) under-5 residing in WAR and WAU who visited the out-patient departments of the selected health care facilities were included.

### 2.3. Sample Size

The sample size was calculated using Cochran’s formula (1977) (pp. 72–76) [16]. Where: *n* = sample size; *z* = z value 1.96 for 95% confidence level; *p* = proportion of malaria prevalence in children under-5 in Sierra Leone [17]; *d* = absolute error allowance/precision. Hence, the sample size was calculated as follow: $1.96^{2}\left[0.205\times \left(1-0.205\right)\right]/0.05^{2}=250$. Meanwhile, 40% of the calculated sample size was added to the study population to account for missing vital data. The final sample size was 350.

(1) $$n=\frac{z^{2}\left[\left(p\right)\times \left(1-p\right)\right]}{d^{2}}$$

### 2.4. Survey Questionnaire

Based on the conceptual framework of this study, we developed a questionnaire that included items mainly referred from previous studies, validated and supplemented according to the biological characteristics of malaria epidemics [18,19]. The questionnaire was written in English, confirmed by pretesting to ensure respondents understood, and thus used for a face-to-face interview. Trained investigators read each question to the participants and explained it in the local language if the participants did not understand English.

The final survey questionnaire contained open and closed-ended questions, and it was divided into four sections (Supplementary file S1). Section A focused on the socio-demographic characteristics of the caregivers. Section B assessed the caregiver’s knowledge of the cause of malaria and its mode of transmission; signs and symptoms of malaria; and prevention. Section C assessed the caregiver’s attitudes toward malaria such as how serious of a health problem they considered malaria to be, if they slept under a bed net with their child(ren), what time of day they thought mosquitoes bite most, and what they thought was the best treatment for malaria. Meanwhile, Section D assessed the caregiver’s practices toward malaria prevention, such as the kind of measures they took to protect their children from mosquito bites, what they did when their child(ren) had a fever or was prescribed an antimalaria medication, what influenced their action and how long they waited before seeking medical care for their febrile child(ren).

### 2.5. Data Collection

After gaining ethical clearance and permission from the Sierra Leone Ethics and Scientific Review Committee, a face-to-face interview with participating caregivers and trained investigators was performed on workdays from mid-August to late-September 2019 using the questionnaire in six selected health care facilities in WAU and WAR.

### 2.6. Statistical Analysis

Socio-demographic characteristics and KAPs data were collected via the face-to face interview questionnaire from 350 caregivers. Descriptive statistics was used to analyze the socio-demographic characteristics of the respondents, and the results were expressed in frequencies and percentages. A scoring system was utilized in the evaluation of the KAPs data (Supplementary file S2) [10,20,21]. KAPs levels acted as dependent variables of the univariate analysis, and they were defined by the 60% cut-off value of the total score. The responses to the KAPs variables between the respondents from WAU and WAR were compared using Chi-square analysis. Fisher’s exact test was used when more than 20% of cell counts were less than five. Spearman’s rank test was used to determine the extent of correlation between KAPs scores since data was not normally distributed as per the outcome of the Kolmogorov-Smirnov test. Based on the rule of thumb of Cohen [22], the strength of correlation was interpreted as 0 = no relationship, 0.10–0.29 = small/low correlation, 0.30–0.49 = medium/moderate correlation, and 0.50–1.00 = large/high correlation.

The association between KAPs and the socio-demographic characteristics of the respondents was evaluated by univariate analyses. All variables significant in the univariate analysis with a *p* ≤ 0.25 were included in the multivariate logistic regression model. Reduced subset models were developed using elimination based on the AIC (Akaike information criteria) score. The level of statistical significance was set at 0.05. All of the data analyses and figure drawing were conducted in R software (Version: 3.6.1).

## 3. Results

### 3.1. Socio-Demographic Characteristics of the Caregivers

As shown in Table 1, all 350 caregivers interviewed completed the survey with a nearly equal number of respondents from WAU and WAR. Most of them were less than 30 year-old mothers. A total of 70.0% of the caregivers were married, while 19.4% were single, and most caregivers were unemployed younger mothers. The total unemployed reached 46.3%, including 33 younger students. In total, 27.1% of them had no educational experience and only 64.3% had at least a secondary level of education. However, all of them had at least one child under-5 living with them and 54.6% had at least one more child above-5.

### 3.2. Knowledge

**Figure 2:**
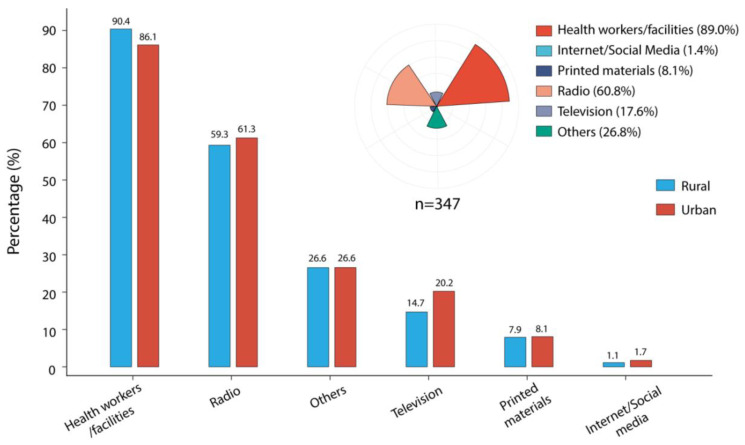
Sources of malaria related information mentioned by caregivers of children under-5. The upper right diagram shows the proportion of each source of malaria related information selected by the caregivers in this study, the different colors represent different sources. The bar plot shows the popularizing rate of the specific source across rural and urban districts, blue and red represent the rural and urban, respectively.

Up to 99.1% of the caregivers had heard of malaria, and no significant difference in malaria related information sources (*p* = 0.858) was found between the caregivers from WAR and WAU (Supplementary file S3: Table S1). Most of them received malaria related knowledge from health workers/facilities (88.3%), followed by radio (60.3%), television (17.4%), and printed materials, such as billboards/handbills (8.0%). Besides, 26.6% received information from other sources, such as community sensitization and announcements about malaria, their friends/peers, and also neighbors; and, only 1.4% were informed through the internet/social media, including only 1.7% of those residing in WAU, where the use of internet/social media was expected to be high—just a little higher than those living in WAR (1.1%) (Figure 2).

**Figure 3:**
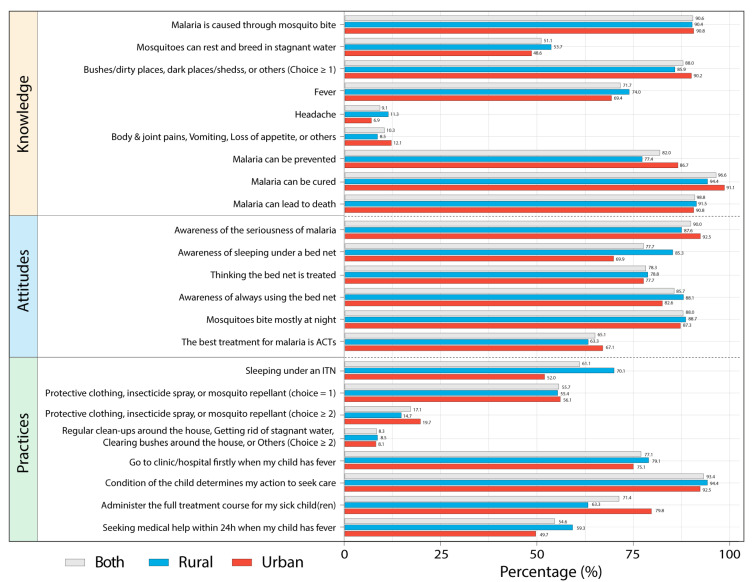
Correct response rates for questions in sections: Knowledge, Attitudes, and Practices. Each item scores 1, except for, “Sleeping under an ITN” in section Practices scoring 2, which were evaluated by the scoring system described in Supplementary file S2.

As displayed in Figure 3 and Supplementary file S3: Table S1, the percentage of respondents that did not know malaria could be prevented and cured or that it could lead to death were 18.0%, 3.4%, and 8.8%, respectively. Among them, only 1.2% of urban caregivers were unaware of the lethality of malaria, whereas the number for rural caregivers was over four times higher (5.6%) (*p* = 0.05). When asked about the symptoms of malaria, the most common responses were fever (71.1%), followed by vomiting (42.9%), loss of appetite (30.9%), and body and joint pains (26.9%), with only a few responses for headaches (9.1%). Approximately 58.6% of them stated other signs and symptoms they knew such as weakness and dizziness, dark colored urine, yellow/pale/white eyes, etc. Approximately 10.0% did not know that malaria parasites can be transmitted through mosquito bites. This study revealed that misconceptions about the causes and the mode of transmission of malaria still prevailed among the caregivers, as 0.3% of them stated that eating too much and having close contacts with a person who has malaria can cause malaria. Furthermore, 4.0% of them stated other misconceptions about the cause of malaria, such as eating too many oranges, not washing hands regularly, cold and flu, dirty hands and feet, flies, drinking too many sweet/soft drinks, etc. While 86.6% of the caregivers stated that bushes/dirty places were convenient resting/breeding places for mosquitoes, followed by stagnant water (51.1%), and dark places/sheds (11.7%).

### 3.3. Attitudes

**Figure 4:**
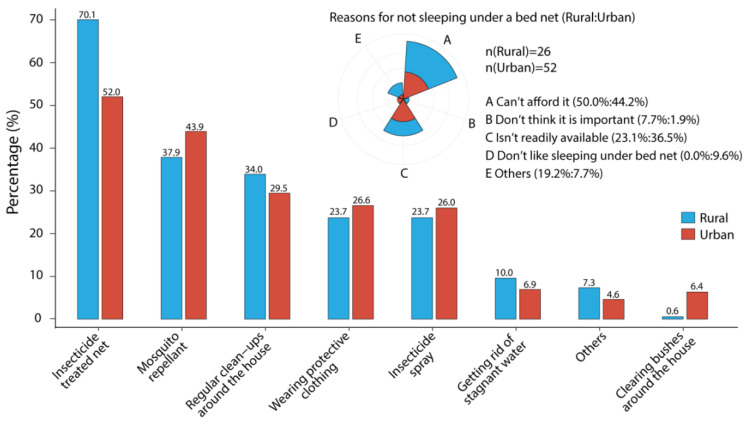
Protective measures against mosquito bites and the reasons for not sleeping under a bed net. The upper right diagram shows the reasons why some caregivers did not use insecticides treated nets (ITNs) in both rural and urban districts, and the proportion of the reason is shown behind the legend as Rural: Urban (%). The bar plot shows the protective measures against mosquito bites employed by caregivers of children under-5 in rural and urban districts. Blue and red represent the rural and the urban, respectively.

As summarized in Figure 3 and Supplementary file S4: Table S2, although 90.0% of respondents believed that malaria was a very serious health problem, 3.1% still said, “No”, 5.4% said, “Not sure”, and 1.4% said, “Don’t know”. A total of 77.7% said that they and their children slept under bed nets, while the rural caregivers (85.3%) had a significantly more positive attitude than the urban (69.9%) (*p* < 0.001). Explaining their children’s lack of bed nets, 46.2% caregivers mentioned that they could not afford them, 32.1% said they were not readily available, 6.4% did not like sleeping under bed nets, 3.8% thought it was not important, and 11.5% stated other reasons such as the statements that they and their children would have allergic reactions or sweat a lot whenever sleeping under bed nets (Supplementary file S4: Table S2, Figure 4). Moreover, when asked about how often their children used the bed net, 85.7% stated always and 14.3% sometimes, but 21.7% did not think the bed net was treated with insecticide. Meanwhile, 88.0% of the respondents thought that malaria-transmitted mosquitoes bite mostly at night. ACTs were considered as the best treatment for malaria by 65.1% caregivers, but 32.0% said, “Don’t know”, and 1.4% also shared other opinions such as injections, intravenous solutions, etc.

### 3.4. Practices

As shown in Figure 3 and Supplementary file S5: Table S3, although 77.1% of respondents would go to a hospital or a clinic if their child had a fever, 19.4% still preferred to first self-medicate at home, and 3.4% would go to a pharmacy first. As stated by 93.4% of the caregivers, the most important factor influencing their action to seek medical help was the condition of the child, followed by perceived cost involved (3.7%), and time availability (1.7%). Furthermore, only 54.6% of the caregivers would seek medical attention for their febrile children within 24 h, whilst 45.1% usually waited for 2–5 days. Meanwhile, when an antimalarial medication had been prescribed, only 71.4% of caregivers mentioned administration of a complete treatment course, but 12.9% stop administration as soon as the child began to show improvement. There was a significantly higher rate among the urban caregivers (79.8%) who performed a complete treatment course than among the rural (63.3%) (*p* < 0.001).

Regarding the use of protective measures against malaria-transmitted mosquitoes, the most common practices employed by 61.1% of respondents was sleeping under ITNs, the rural caregivers had a significantly higher rate of ITNs use than the urban (70.1% vs. 52.0%, *p* < 0.01) (Figure 4). However, we observed that misuse of bed nets, such as using them for doing pond fishing and covering backyard gardens, is still common—especially in rural and provincial areas of Sierra Leone (Supplementary file S6: Figure S1). Other common selections for protection included using mosquito repellant (40.9%), regularly cleaning the surroundings of the house (31.7%), wearing protective clothing (25.1%), and using insecticide spray (24.9%). Only a few caregivers said that they got rid of stagnant water (8.3%) and cleared bushes around the house (3.4%), with 11 (6.4%) of them residing in WAU and only 1 from WAR (0.6%). Additionally, 6.0% of caregivers mentioned other protective measures, such as closing the windows and the doors early before dark, fixing a mesh on window and door frames to keep mosquitoes out, etc., (Figure 3 and Figure 4, Supplementary file S5: Table S3).

### 3.5. Correlations among Knowledge, Attitudes, and Practices

Inferred by the Spearman correlation test (Table 2), a significant positive correlation was found between knowledge-attitudes (r_s_ = 0.13, *p* < 0.05) and attitudes-practices (r_s_ = 0.45, *p* < 0.001). Further univariate logistic regression analysis indicated that improved knowledge might positively affect attitudes (OR = 1.98; 95% CI = 1.21–3.25), while positive attitudes might lead to good practices (OR = 7.91; 95% CI = 4.33–15.54).

### 3.6. Effects of Socio-Demographic Characteristics on KAPs

Through univariate analysis, the association of socio-demographic and malaria-related KAPs were demonstrated by comparing with referred groups (Table 3). The dependent variable of the univariate analysis is the levels of KAPs, which was scored and categorized into two levels according to the designed scoring system (Supplementary file S7: Table S4). The age, religion, educational background, and occupation were found to correlate with knowledge. The caregivers in the >30 year-old group tended to have higher knowledge than the 15–20 year-old group (OR = 3.03, 95% CI = 1.35–7.28). In comparison to the Muslim caregivers, Christian faith was positively associated with knowledge (OR = 2.20, 95% CI = 1.29–3.86). The caregivers with a secondary education had higher knowledge than the group with no education (OR = 1.94, 95% CI = 1.15–3.28). Meanwhile, compared with the unemployed caregivers, students knew more malaria-related information (OR = 3.04, 95% CI = 1.20–9.33) but represented a negative practice (OR = 0.22, 95% CI = 0.07–0.56). As for attitudes, age, educational background, and district (urban or rural) were inferred to be associated. A positive association with attitudes was found in the respondents with a university education (OR = 4.46, 95% CI = 1.42–19.73) and the rural caregivers (OR = 1.83, 95% CI = 1.14–2.96), but a negative association was found in the caregivers of the 21–25 year-old group (OR = 0.39, 95% CI = 0.19–0.76).

Furthermore, independent predictors of KAPs were inferred by a multivariate logistic regression analysis comparing referred groups (Table 4). Age, religion, education, and occupation were found to be independent predictors for malaria-related knowledge. The following groups were more likely to have a higher knowledge of malaria: 26–30 year olds (OR = 2.14, 95% CI = 1.02–4.55), >30 year olds (OR = 4.83, 95% CI = 1.95–12.69), caregivers with a secondary education (OR = 2.34, 95% CI = 1.32–4.19), Christians (OR = 2.28, 95% CI = 1.30–4.14), and students (OR = 2.98, 95% CI = 1.12–9.48). Meanwhile, district, education, age, and relationship were found to be independent predictors of malaria-related attitudes. Rural caregivers (OR = 1.97, 95% CI = 1.20–3.28) and the group with a higher education (university level, OR = 5.53, 95% CI = 1.67–25.54; secondary level, OR = 1.86, 95% CI = 1.05–3.30), were more likely to have a positive attitude toward malaria, while caregivers in the 21–25 year-old group (OR = 0.40, 95% CI = 0.19–0.79) and fathers (OR = 0.19, 95% CI = 0.04–0.80) were more likely to have negative malaria-related attitudes. Meanwhile the relationship, occupation, and marital status were taken as independent predictors for malaria-related practices. The mothers seemed more likely to have better practices toward malaria control and prevention as compared to the fathers (OR = 0.19, 95% CI = 0.04–0.80). The caregivers who were students (OR = 0.11, 95% CI = 0.01–0.55), married (OR = 0.53, 95% CI = 0.29–0.97), or in a consensual union (OR = 0.30, 95% CI = 0.12–0.74) were more likely to have bad malaria-related practices.

## 4. Discussion

### 4.1. Knowledge

In our study, the respondents in the Western Area in Sierra Leone received malaria related information mainly from health workers/facilities followed by radio as similar to previous studies [11,18], while 26.6% of respondents named other sources of information, such as community sensitization, meetings, and announcements, in accordance with the study conducted in Swaziland [11]. Only a few caregivers mentioned TV and printed materials (billboards/handbills, etc.) as sources, even fewer received the information from the internet or from social media, which might be due to limited accessibility to, and the high cost of, internet technologies in Sierra Leone. However, in Saudi Arabia, the main source of malaria related information was recorded from the internet and social media [12]. Additionally, even when confronted with very limited financial and human resources for healthcare in the 1950s in China, the cooperation among different sectors and paramedics, such as barefoot doctors at the village level, played a very important role in schistosomiasis control programs [23]. As for our references, therefore, more effort in Sierra Leone should be given to enhance caregivers’ knowledge by continuously improving the content, type, and media of malaria related messages so as to facilitate the establishment of an effective community-coordinated health-education network and thereby achieve proper health education coverage for various groups in the urban and the rural regions. A highly effective mean could be developed by training paramedics at the community level using audio–visual messages in simple local languages for the caregivers to facilitate communication, especially for those not, or under, educated. In addition, the media infrastructure of the communities such as the radio, TV, and internet, or social media platforms, also need to be gradually improved upon in the course of local socio-economic development.

With regard to malaria, fever is the highest known symptom followed by other symptoms, such as weakness, dizziness, dark colored urine, etc., which were mostly mentioned by the respondents. Fever was recorded as the most known and common symptom of malaria in children under-5 in Cabo Verde and Zambia [2,13]. Other symptoms such as anemia were most associated with malaria in children under-5 according to reports in Tanzania [24,25]. Similar to other surveys [26,27], our study revealed that approximately 90% of the respondents knew malaria was transmitted through the bite of an infected mosquito. However, some misconceptions about the cause and the transmission of malaria was mentioned by some caregivers, such as eating too many oranges, not washing hands regularly, catching a cold and flu, dirty hands and feet, flies, drinking too many sweet/soft drinks, etc. The other misconceptions of malaria transmission, including prolonged sun exposure and drinking dirty water, were also reported in Tanzania and Uganda [24,28]. Regarding the resting and the breeding places of mosquitoes, bushes/dirty places and stagnant water were the most frequently mentioned by the respondents, which is consistent with previous study in Tanzania [29].

### 4.2. Attitudes

In the present study, malaria was known as a very serious health problem by 90% of the caregivers, similar to previous surveys in south-western Saudi Arabia and in Rwanda [30,31]. That malaria-related mosquitoes bite mostly at night time was recognized by 88% of the respondents. This kind of attitude is expected to influence the caregivers to be more vigilant, especially at night, in protecting their children from mosquito bites, but the most important thing was to get rid of mosquito breeding sites and to sleep under an ITN. Recent studies [32,33] agree the idea about the time-of-day of blood-feeding and malaria transmission by mosquitoes being mostly at night time. Compared with an earlier report of the Sierra Leone Demographic Health Survey (SLDHS) in 2013 that only 49.5% of children under-5 slept under a bed net at night [34], our findings showed that 77.7% of the respondents together with their children slept under bed nets. Yet, 78 caregivers still claimed that they did not use the bed net, 46 among them stating that they could not afford it. These are most likely individuals who are socioeconomically disadvantaged, and they are probably waiting for donation campaigns of the usual mass mosquito nets, as some even mentioned that they were not supplied with bed nets at their community health centers or claimed that mosquito nets were not readily available, which means they could probably afford it but could not easily find one to purchase. In essence, some of these common barriers to the use of bed nets have also been discovered by other studies [35,36]. Moreover, 21.7% of the caregivers still do not know anything about ITNs. Meanwhile, ACTs are recommended by the WHO, provided by the health facilities of governments, and even sold in pharmacies and drug stores nationwide as a part of the first-line anti-malaria treatment policy in Sierra Leone [9,37]. Yet, 32% of the caregivers said that they did not know ACTs are the best treatment for the disease.

### 4.3. Practices

Every respondent in this study stated that they employed at least one method of prevention against mosquito bites, with the highest being sleeping under an ITN, followed by using mosquito repellants, and regularly doing clean-up around the house, in accordance with the records in Kenya [38] and Zimbabwe [39]. The other preventive measures, such as wearing protective clothing, using insecticide sprays, getting rid of stagnant water, clearing bushes around the houses, and using mosquito barrier nets on windows and doors, etc., mentioned by the caregivers as good and effective strategies for vector control, are in line with previous study [40].

A good anti-malaria practice is also supported by previous studies [41,42], which have shown that 77% of caregivers come to the hospital first when the child has a fever. From the epidemiological and clinical perspective, fever is not only a crucial indicator of malaria, especially in children, but it may even be the most important warning indicator for administering treatment as recommended by WHO and the Sierra Leone NMCP, especially for the government to offer free health care services for children under-5 [43]. However, as a priority, some caregivers preferred self-medicating or going to a pharmacy when their child presented a fever, which is a dangerous practice that predisposes the sick child to a possibly worse situation. Other studies in Uganda [41] and Myanmar [44] have also found poor care-seeking behaviors for fever cases in children under-5. In our study, furthermore, more than 93% of the caregivers were influenced to seek appropriate medical care upon the conditions of their children, and some of the caregivers also mentioned during further explanations that they would decide to go with their child(ren) to the hospital only if they saw no improvement after self-medicating and observation for a few days. A similar finding was observed in a study done in Sudan, where some parents postponed seeking medical care for their child(ren) for a few days causing the child(ren)’s conditions to deteriorate by the time of going to a medical facility [45]. Nevertheless, our findings showed that approximately 54.6% of the caregivers did seek appropriate medical care within 24 h for their feverish child(ren). As follow-up therapeutic care, 71.4% of the caregivers completed a full course of antimalarial medications as well as the follow-ups, which is very important in maximizing antimalarial efficacy with respect to dosing regimens and preventing relapse. However, 12.9% suspended the administration of antimalarials as soon as their child(ren) showed a little improvement in their clinical symptoms and signs and, surprisingly, some even said they administered the medication to other siblings, all of which are considered harmful practices. Another critical point worth our attention is that previous studies [46] have confirmed the resistance to ACTs in some parts of the world such as South-East Asia, and they have also pointed out that the reduced efficacy has raised major concerns about malaria treatment and control. For this reason, it is essential to follow correct dosing regimens as prescribed in order to limit the spread of antimalarial resistance.

### 4.4. Correlation among Malaria Related KAPs

Although some studies have found that high knowledge about prevention is poorly reflected in practice [11,47], some other studies [48,49] have found that a high or an increased knowledge of malaria and its mode of transmission and infection could promote and improve preventative practices in communities where malaria is highly prevalent. Our findings indicated that good knowledge on malaria might lead to positive attitudes, and better attitudes might facilitate good practices against the disease.

### 4.5. Correlation between Caregivers’ Socio-Demographic Characteristics and KAPs

The majority of the caregivers of children under-5 in this study were young mothers (˂30 year-old), including 33 younger students, and 27.1% of them lacked any educational experience. Financially, most of them were unemployed and underprivileged: even among the 44.3% employed caregivers, some were self-employed, mostly engaged in petty trading and low income businesses from which they could hardly earn enough to meet their basic daily needs. For a relatively immature parent that is socioeconomically handicapped, having the right malaria related KAPs might make up for their deficiency in maturity and their socioeconomic disadvantages [50]. However, being a mother did result in more positive attitudes and practices toward malaria prevention and control than being a father, which supports why only women were engaged in most previously conducted studies on malaria perception or prevention and attitudes [51,52]. Meanwhile, having at least a secondary level of education was found to be associated with a positive influence on malaria related attitudes and knowledge, which is consistent with previous studies [10,53]. Yet, in Zambia, Nzooma et al. [14] reported that higher levels of education were not related to higher knowledge levels and good practices toward malaria control. Our findings indicated that the student mothers might know more but do less.

Moreover, only 1.2% of urban caregivers expressed their unawareness of the lethality of the disease, whereas the number for rural caregivers was over four times higher (5.6%). It is interesting to note that significantly more urban caregivers (79.8%) followed a complete malaria treatment course of ACTs than the rural (63.3%). However, in view of using ITNs for malaria prevention, the rural caregivers (85.3%) presented significantly more positive attitudes than the urban (69.9%), and the rural (70.1%) therefore represented better practices than the urban (52.0%).

## 5. Conclusions

The present KAPs survey toward malaria prevention and control indicates, to some extent, that misconception of the cause, transmission, and clinical symptoms, unawareness of the lethality and severity, and inappropriate behaviors in prevention and treatment, including self-medicating, are still in existence among caregivers of children under-5 in the Western Area of Sierra Leone. The KAPs toward malaria are affected by caregivers’ educational background, occupational status, parenthood, living region, and religion. Positive correlations in knowledge-attitudes and attitudes-practices are evidently demonstrated. Therefore, in order to better protect children under-5 against severe and deadly malaria, their caregivers, especially young mothers, need such prior supports as increased education and employment opportunities. Meanwhile, there is also a need to set up an accessible community-based counselling service toward malaria and to construct an effective community-based communication channel, especially for caregivers of children under-5. The present study may facilitate the implementation of integrated malaria control strategies, especially dealing with malaria infections in children under-5.

## 6. Limitations of this Study

Despite its efficacy in enhancing the understanding of KAPs toward malaria, the present cross-sectional survey did not investigate the cause and the effect of malaria in children under-5. Meanwhile, the survey was conducted by self-reporting, which may cause discrepancies between the reported behaviors, participants’ responses, and actual behaviors due to interviewer bias, participant bias, or response bias. Finally, another possible bias may be derived from the selection of the chosen hospitals or health centers.

## Figures and Tables

**Table 1:** Socio-demographics of caregivers of children under-5 (*n* = 350).

Items	Variables	Frequency	Percentage (%)
Age (years)	15–20	68	19.4
	21–25	128	36.6
	26–30	95	27.1
	>30	59	16.9
Gender	Male	10	2.9
	Female	340	97.1
Religion	Christian	111	31.7
	Muslim	239	68.3
Relationship	Mother	324	92.6
	Father	10	2.9
	Others ^a^	16	4.6
Children under-5	=1	218	62.3
	≥2	132	37.8
Other children above-5	=0	159	45.4
	≥1	191	54.6
District	Urban	173	49.4
	Rural	177	50.6
Marital status	Single	68	19.4
	Married	245	70.0
	Consensual union	37	10.6
Education	None	95	27.1
	Primary ^b^	30	8.6
	Secondary ^c^	198	56.6
	University	27	7.7
Occupational status	Unemployed	162	46.3
	Employed	155	44.3
	Student	33	9.4

^a^ Caregivers have other relationship with the children under-5 including aunt, uncle, and grandparent; ^b^ Caregivers with educational year between 1 and 6 years; ^c^ Caregivers with educational year between 7 and 12 years.

**Table 2:** Relationship between knowledge, attitudes, and practices (*n* = 350).

Variables	Spearman Rank Test		Univariate Logistic Regression Analysis	
	r_s_	*p*	OR (95% CI)	*p*
Knowledge-Attitudes	0.13	0.019 *	1.98 (1.21–3.25)	0.007 **
Knowledge-Practices	0.05	0.333	1.19 (0.74–1.90)	0.476
Attitudes-Practices	0.45	0.000 ***	7.91 (4.33–15.54)	0.000 ***

r_s_: Spearman rank correlation coefficients. CI: Confidence intervals. * *p* < 0.05; ** *p* < 0.01; *** *p* < 0.001.

**Table 3:** Association of socio-demographic characteristics and KAPs scores of caregivers of children under-5 (*n* = 350).

Variables	Knowledge		Attitudes		Practices	
	OR (95% CI)	*p*	OR (95% CI)	*p*	OR (95% CI)	*p*
Age (Years) (Group Age of 15–20 as the reference)						
21–25	1.31 (0.71–2.42)	0.384	0.39 (0.19–0.76)	0.007 **	0.90 (0.49–1.63)	0.715
26–30	1.73 (1.25–8.11)	0.107	1.04 (0.47–2.24)	0.927	1.00 (0.54–1.88)	0.991
>30	3.03 (1.35–7.28)	0.009 **	0.83 (0.36–1.94)	0.670	1.00 (0.49–2.02)	0.995
Gender (Male as the reference)						
Female	-	-	2.66 (0.72–9.75)	0.129	7.11 (1.11–111.28)	0.064
Religion (Group Muslim as the reference)						
Christian	2.20 (1.29–3.86)	0.005 **	1.63 (0.97–2.80)	0.072	0.73 (0.46–1.15)	0.173
Relationship (Mother as the reference)						
Father	-	-	0.36 (0.10–1.33)	0.114	0.14 (0.01–0.74)	0.061
Others	1.91 (0.60–8.45)	0.322	0.47 (0.17–1.34)	0.140	0.56 (0.17–1.58)	0.294
Children under-5 (Group having 1 child under-5 as the reference)						
≥2	1.03 (0.64–1.66)	0.909	1.06 (0.66–1.73)	0.814	1.49 (0.96–2.31)	0.074
Children above-5 (Group having no child above-5 as the reference)						
≥1	0.83 (0.52–1.32)	0.431	1.09 (0.68–1.74)	0.723	1.13 (0.74–1.73)	0.574
District (Group Urban as the reference)						
Rural	1.15 (0.73–1.83)	0.544	1.83 (1.14–2.96)	0.012 *	1.24 (0.81–1.90)	0.317
Marital status (Group Single as the reference)						
Married	0.70 (0.36–1.28)	0.257	1.05 (0.57–1.89)	0.869	0.87 (0.51–1.50)	0.619
Consensual union	0.73 (0.30–1.82)	0.488	0.72 (0.30–1.71)	0.445	0.54 (0.23–1.23)	0.149
Education (Group No education as the reference)						
Primary	1.10 (0.48–2.64)	0.823	2.23 (0.87–6.50)	0.112	0.68 (0.29–1.55)	0.366
Secondary	1.94 (1.15–3.28)	0.013 *	1.45 (0.86–2.44)	0.164	0.71 (0.43–1.16)	0.167
University	2.81 (1.04–8.96)	0.055	4.46 (1.42–19.73)	0.021 *	0.70 (0.29–1.66)	0.424
Occupational status (Group Unemployed as the reference)						
Employed	1.56 (0.97–2.54)	0.071	0.97 (0.59–1.59)	0.908	1.14 (0.73–1.78)	0.556
Student	3.04 (1.20–9.33)	0.030 *	0.75 (0.34–1.71)	0.474	0.22 (0.07–0.56)	0.003 **

OR: Odds ratio. CI: Confidence intervals. * *p* < 0.05; ** *p* < 0.01.

**Table 4:** Multivariate logistic regression analysis of KAPs (*n* = 350).

KAPs	Variables	OR (95% CI)	*p*
Knowledge	Age (Years) (Group Age of 15–20 as the reference)		
	21–25	1.44 (0.74–2.80)	0.277
	26–30	2.14 (1.02–4.55)	0.046 *
	>30	4.83 (1.95–12.69)	0.001 ***
	Religion (Group Muslim as the reference)		
	Christian	2.28 (1.30–4.14)	0.005 **
	Education (Group No education as the reference)		
	Primary	0.99 (0.41–2.45)	0.978
	Secondary	2.34 (1.32–4.19)	0.004 **
	University	1.55 (0.53–5.25)	0.445
	Occupation status (Group Unemployed as the reference)		
	Employed	1.26 (0.75–2.12)	0.391
	Student	2.98 (1.12–9.48)	0.041 *
Attitudes	Age (Years) (Group Age of 15–20 as the reference)		
	21–25	0.40 (0.19–0.79)	0.01 **
	26–30	1.03 (0.45–2.32)	0.94
	>30	1.12 (0.44–2.89)	0.814
	District (Group Urban as the reference)		
	Rural	1.97 (1.20–3.28)	0.008 **
	Education (Group No education as the reference)		
	Primary	2.26 (0.83–7.08)	0.132
	Secondary	1.86 (1.05–3.30)	0.034 *
	University	5.53 (1.67–25.54)	0.011 *
	Relationship (Mother as the reference)		
	Father	0.19 (0.04–0.80)	0.022 *
	Others	0.48 (0.16–1.47)	0.187
Practices	Relationship (Mother as the reference)		
	Father	0.11 (0.01–0.60)	0.037 *
	Others	0.60 (0.17–1.88)	0.396
	Religion (Group Muslim as the reference)		
	Christian	0.64 (0.39–1.06)	0.083
	Occupational status (Group Unemployed as the reference)		
	Employed	1.44 (0.90–2.30)	0.127
	Student	0.22 (0.07–0.59)	0.005 **
	Marital status (Group Single as the reference)		
	Married	0.53 (0.28–0.97)	0.043 *
	Consensual union	0.37 (0.15–0.89)	0.029 *
	Children under-5 (Group having 1 child under-5 as the reference)		
	≥2	1.46 (0.92–2.34)	0.109

OR: Odds ratio. CI: Confidence intervals. * *p* < 0.05; ** *p* < 0.01; *** *p* < 0.001.

## Data Availability

The datasets used and analyzed in the study are available from the corresponding author on reasonable request.

## Supplementary Materials

The following supporting information can be downloaded at: https://www.mdpi.com/article/10.3390/tropicalmed7070120/s1, Supplementary file S1: KAPs questionnaire designed for the caregivers of children under-5; Supplementary file S2: Scoring system; Supplementary file S3: Table S1: Comparison of knowledge toward malaria between caregivers living in rural and urban districts; Supplementary file S4: Table S2: Comparison of attitudes toward malaria between caregivers living in rural and in urban districts; Supplementary file S5: Table S3: Comparison of practices toward malaria between caregivers living in rural and in urban districts; Supplementary file S6: Figure S1: Images of mosquito bed net misuse in Sierra Leone; Supplementary file S7: Table S4: KAPs levels of caregivers toward malaria control/prevention based on the scoring system.

## References

[1] World Health Organization (2021). World Malaria Report 2021.

[2] DePina A.J., Dia A.K., Martins A., Ferreira M.C., Moreira A.L., Leal S.V., Pires C.M., Moreira J.M.G., Tavares M.F., da Moura A.J.F. (2019). Knowledge, attitudes and practices about malaria in Cabo Verde: A country in the pre-elimination context. BMC Public Health.

[3] Government of Sierra Leone Ministry of Health and Sanitation National Malaria Control Programme (2015). Guidelines for Case Management of Malaria in Sierra Leone.

[4] NMCP, INFORM, LSHTM (2015). Sierra Leone: A Profile of Malaria Control and Epidemiology.

[5] Tabbabi A. (2018). Socio-economic impact of malaria in Africa. ASMI.

[6] Government of Sierra Leone Ministry of Health and Sanitation (2015). Sierra Leone Malaria Control Strategic Plan 2016–2020.

[7] World Health Organization (2017). A Framework for Malaria Elimination.

[8] World Health Organization (2021). Global Technical Strategy for Malaria 2016–2030, 2021 Update.

[9] Catholic Relief Services (CRS), College of Medicine and Allied Health Sciences, University of Sierra Leone (COMAHS), ICF International, National Malaria Control Programme (Sierra Leone), Roll Back Malaria Partnership, Statistics Sierra Leone (2017). Sierra Leone Malaria Indicator Survey 2016.

[10] Oladimeji K.E., Tsoka-Gwegweni J.M., Ojewole E., Yunga S.T. (2019). Knowledge of malaria prevention among pregnant women and non-pregnant mothers of children aged under 5 years in Ibadan, South West Nigeria. Malar. J..

[11] Hlongwana K.W., Mabaso M.L., Kunene S., Govender D., Maharaj R. (2009). Community knowledge, attitudes and practices (KAPs) on malaria in Swaziland: A country earmarked for malaria elimination. Malar. J..

[12] Khairy S., Al-Surimi K., Ali A., Shubily H.M., Walaan N.A., Househ M., Walaan N.A., Househ M., El-Metwally A. (2017). Knowledge, attitude and practice about malaria in south-western Saudi Arabia: A household-based cross-sectional survey. J. Infect. Public Health.

[13] Yaya S., Bishwajit G., Ekholuenetale M., Shah V., Kadio B., Udenigwe O. (2017). Knowledge of prevention, cause, symptom and practices of malaria among women in Burkina Faso. PLoS ONE.

[14] Shimaponda-Mataa N.M., Tembo-Mwase E., Gebreslasie M., Mukaratirwa S. (2016). Knowledge, attitudes and practices in the control and prevention of malaria in four endemic provinces of Zambia. SAJID.

[15] Kassam R., Sekiwunga R., MacLeod D., Tembe J., Liow E. (2016). Patterns of treatment-seeking behaviors among caregivers of febrile young children: A Ugandan multiple case study. BMC Public Health.

[16] Cochran W.G. (1977). Sampling Techniques.

[17] U.S. President’s Malaria Initiative (2020). Sierra Leone Malaria Operational Plan.

[18] Gupta R.K., Raina S.K., Shora T.N., Jan R., Sharma R., Hussain S. (2016). A household survey to assess community knowledge, attitude and practices on malaria in a rural population of Northern India. JFMPC.

[19] Janet K., Catherine A., Folake O., Precious O., Yewande D.A. (2016). Knowledge, attitudes and practices of mothers of under-five regarding prevention of malaria in children: Evidence from ogun state, Nigeria. IOSR-JHSS.

[20] Onarheim S.A., Andrew Y.K., Bjørn B. (2014). Education and knowledge helps combating malaria, but not degedege: A cross-sectional study in Rufiji, Tanzania. Malar. J..

[21] WHO: Malaria Fact Sheet. https://www.who.int/news-room/fact-sheets/detail/malaria.

[22] Cohen J. (1988). Statistical Power Analysis for the Behavioral Sciences.

[23] Collins C., Xu J., Tang S. (2012). Schistosomiasis control and the health system in P.R. China. Infect. Dis. Poverty.

[24] Mazigo H.D., Obasy E., Mauka W., Manyiri P., Zinga M., Kweka E.J., Mnyone L.L., Heukelbach J. (2010). Knowledge, attitudes, and practices about malaria and its control in rural northwest Tanzania. Malar. Res. Treat..

[25] Smithson P., Florey L., Salgado S.R., Hershey C.L., Masanja H., Bhattarai A., Mwita A., McElroy P.D. (2015). Tanzania Malaria Impact Evaluation Research Group. Impact of malaria control on mortality and anemia among Tanzanian children less than five years of age, 1999–2010. PLoS ONE.

[26] Nejati J., Moosa-Kazemi S.H., Saghafipour A., Soofi K. (2018). Knowledge, attitude and practice (KAP) on malaria, from high malaria burden rural communities, southeastern Iran. J. Parasit. Dis..

[27] Govere J., Durrheim D., Grange K., Mabuza A., Booman M. (2000). Community knowledge and perceptions about malaria and practices influencing malaria control in Mpumalanga Province, South Africa. S. Afr. Med. J..

[28] Obol J., Lagoro K., Garimoi O.C. (2011). Knowledge and misconceptions about malaria among pregnant women in a post-conflict internally displaced persons’ camps in Gulu district, northern Uganda. Malar. Res. Treat..

[29] Mathania M.M., Kimera S.I., Silayo R.S. (2016). Knowledge and awareness of malaria and mosquito biting behaviour in selected sites within Morogoro and Dodoma regions Tanzania. Malar. J..

[30] Dujing S.L. (2015). Malaria in Children under 5 Years in the Gushegu District Hospital before and after Indoor Residual Spraying in Northern Ghana. Master’s Thesis.

[31] Nyirakanani C., Chibvongodze R., Habtu M., Masika M., Mukoko D., Njunwa K.J. (2018). Prevalence and risk factors of asymptomatic malaria among under-five children in Huye District, Southern Rwanda. Tanzan. J. Health Res..

[32] O’Donnell A.J., Rund S.S.C., Reece S.E. (2019). Time-of-day of blood-: Effects on mosquito life history and malaria transmission. Parasites Vectors.

[33] Pigeault R., Caudron Q., Nicot A., Rivero A., Gandon S. (2018). Timing malaria transmission with mosquito fluctuations. Evol. Lett..

[34] Statistics Sierra Leone (SSL) and ICF International (2014). Sierra Leone Demographic and Health Survey 2013.

[35] Htwe E.P. (2017). Caregivers’ Malaria Preventive Practices for Under-Five Children and Its Association in Ngapudaw High-Risk Township, Ayeyarwady Region-Myanmar. Master’s Thesis.

[36] Lungu E.A., Obse A.G., Darker C., Biesma R. (2018). What influences where they seek care? Caregivers’ preferences for under-five child healthcare services in urban slums of Malawi: A discrete choice experiment. PLoS ONE.

[37] WHO (2015). Guidelines for the Treatment of Malaria.

[38] Taylor C., Florey L., Ye Y. (2017). Equity trends in ownership of insecticide-treated nets in 19 sub-Saharan African countries. Bull. World Health Organ..

[39] Tizifa T.A., Kabaghe A.N., McCann R.S., van den Berg H., Van Vugt M., Phiri K.S. (2018). Prevention efforts for malaria. Curr. Trop. Med. Rep..

[40] Gachelin G., Garner P., Ferroni E., Verhave J.P., Opinel A. (2018). Evidence and strategies for malaria prevention and control: A historical analysis. Malar. J..

[41] Mpimbaza A., Ndeezi G., Katahoire A., Rosenthal P.J., Karamagi C. (2017). Demographic, socioeconomic, and geographic factors leading to severe malaria and delayed care seeking in Ugandan children: A case-control study. Am. J. Trop. Med. Hyg..

[42] Adinan J., Damian D.J., Mosha N.R., Mboya I.B., Mamseri R., Msuya S.E. (2017). Individual and contextual factors associated with appropriate healthcare seeking behavior among febrile children in Tanzania. PLoS ONE.

[43] Measure Evaluation (2019). Capacity of Sierra Leone’s National Malaria Control Programme for Monitoring and Evaluation: Baseline Assessment.

[44] Thandar M.M., Kyaw M.P., Jimba M., Yasuoka J. (2015). Caregivers’ treatment-seeking behaviour for children under age five in malaria-endemic areas of rural Myanmar: A cross-sectional study. Malar. J..

[45] Elfaki A.E.M., Elnimeiri M.K.M., Elfakey W.E.M. (2017). Management seeking behavior of malaria among mothers of under-five year’s children in Damazin Locality, Blue Nile State, Sudan. IJPSI.

[46] Beeson J.G., Boeuf P., Fowkes F.J. (2015). Maximizing antimalarial efficacy and the importance of dosing strategies. BMC Med..

[47] Singh R.K., Haq S., Dhiman R.C. (2013). Studies on knowledge, attitude and practices in malaria endemic tribal areas of Bihar and Jharkhand, India. J. Trop. Dis. Public Health.

[48] Israel O.K., Fawole O.I., Adebowale A.S., Ajayi I.O., Yusuf O.B., Oladimeji A., Ajumobi O. (2018). Caregivers’ knowledge and utilization of long-lasting insecticidal nets among under-five children in Osun State, Southwest, Nigeria. Malar. J..

[49] Eseigbe E.E., Anyiam J.O., Ogunrinde G.O., Wammanda R.D., Zoaka H.A. (2012). Health care seeking behavior among caregivers of sick children WHO had cerebral malaria in northwestern Nigeria. Malar. Res. Treat..

[50] Ricci F. (2012). Social implications of malaria and their relationships with poverty. Mediterr. J. Hematol. Infect. Dis..

[51] Davies M. (2018). Women’s Perceptions of Malaria in the Western Rural Areas of Sierra Leone. Ph.D. Thesis.

[52] Orimadegun A.E., Ilesanmi K.S. (2015). Mothers’ understanding of childhood malaria and practices in rural communities of Ise-Orun, Nigeria: Implications for malaria control. J. Fam. Med. Prim. Care.

[53] Mitiku I., Assefa A. (2017). Caregivers’ perception of malaria and treatment-seeking behaviour for under five children in Mandura District, West Ethiopia: A cross-sectional study. Malar. J..

