# Supplementary material for: A Cross-Sectional Survey on the Malaria Control and Prevention Knowledge, Attitudes, and Practices of Caregivers of Children Under-5 in the Western Area of Sierra Leone
Source: Trop Med Infect Dis. 2022 Jun 28;7(7):120. doi: 10.3390/tropicalmed7070120 (PMC9319430; doi:10.3390/tropicalmed7070120)
Supplement: Supplementary file 1 [file tropicalmed-07-00120-s001.zip › Supplementary file S1.pdf]

Informed Consent

Dear Participant, my name is Joan Mabinty Koroma, a Sierra Leonean and student of Southern Medical University in P.R. China. I am conducting a survey to assess the knowledge, attitudes and practices among caregivers and the prevalence of malaria in children under 5 in Western Area of Sierra Leone. I am kindly asking that you answer questions for this survey to enable collection of data. The interview will take about ten (10) minutes of your time. All the information obtained from your response will remain strictly confidential and private. Also, you are not obliged to answer any question you do not want to, and you may stop the interview at any time without any penalty.

Do you agree to participate? Yes ___ No___ Signature/thumb print_________

Participant code: _______________________ Date_______________________

A. Socio-demographics (Caregiver)

1. Age (in years) __________ (Please estimate if unknown)

2. Sex: M ( ) F ( )

3. Religion: Christian( ) Muslim( ) Others(please specify)_______________

4. What is your relationship with the child you take care of:
   Mother( ) Father( ) Aunt( ) Uncle( )
   Others(please specify)_________________

5. How many children do you currently have living with you under the age of 5? ______

6. How many other children do you have? __________

7. Where do you live? Urban( ) Rural( )
   Detailed Address____

8. Marital status: Married( ) Single( ) Separated( ) Divorced( )
   Consensual union [living together]( ) Widow/Widower( )

9. Educational level: None( ) Primary( ) Secondary( )
   Tech-Voc( ) University( )

10. Occupational Status**:** Government-sector employed( ) Private-sector employed( )
    Self-employed( ) Unemployed( ) Student( ) Retired( )

B. Demographics of child/ children under-5

	Child's sex	Child's age (in years/months) (Please estimate if unknown)
Child 1		
Child 2		
Child 3		

C. Knowledge about malaria

1. Have you ever heard of malaria?

Yes ( ) No ( )

2. Source of information about malaria: [Tick all that apply]
   TV ( ) Internet/ social media ( ) Radio ( ) Health workers/facilities ( )
   Printed material [billboards/handbills/magazines/newspapers] ( )
   Others____

3. What causes malaria?
   Parasite through mosquito bites ( ) Eating too much ( )
   Close contact with a person who has malaria ( ) Don't know ( )
   Others (specify)____

4. Resting and breeding places of mosquitoes: [Tick all that apply]
   Stagnant water ( ) Bushes/dirty places ( ) Dark places/sheds ( )
   Don't know ( ) Others (specify)__________________________

5. What signs & symptoms of malaria are you aware of? [Tick all that apply]
   Fever ( ) Headache ( ) Body and joint pains ( )
   Vomiting ( ) Loss of appetite ( ) Don't know ( )
   Others____

6. Can malaria be prevented? Yes ( ) No ( ) Don't know ( )

7. Can malaria be cured? Yes ( ) No ( ) Don't know ( )

8. Can malaria lead to death? Yes ( ) No ( ) Don't know ( )

D. Attitude towards malaria

1. Do you think malaria is a very serious health problem?
   Yes ( ) No ( ) Not sure ( ) Don't know ( )

2. Do you and your child(ren) sleep under a bed net? **[if YES go to Q3, if NO go to Q5]**
   Yes ( ) No ( )

3. If Yes, is the bed net:
   Treated ( ) Not treated ( ) Don't know ( )

4. How often do you and your child(ren) use the bed net?
   Always ( ) Sometimes ( ) Never ( )

5. If NO, why?

   I can't afford it ( )

   It is not readily available ( )

   My child(ren) and I don't like sleeping under a bed net ( )

   I don't think it is important ( )

   Others____

6. At what time of day do you think mosquitoes bite the most?

   Daytime ( ) Night time ( )

   Any time ( ) Don't know ( )

7. What do you think is the best treatment for malaria?

   ACTs ( ) Paracetamol ( )

   Traditional/Herbal medications ( ) Don't know ( )

   Others___________________________________

E. Practice

1. What measures do you take to protect you and your child(ren) from mosquito bites?

   [Tick all that apply]:

   Insecticide spray ( ) Mosquito repellant ( )

   Wearing protective clothing ( ) Clearing bushes around the house ( )

   Getting rid of stagnant water ( ) Sleeping under insecticide-treated bed net ( )

   Regular clean-ups around the house ( ) Others__________________________

2. What is the first thing you do when your child(ren) has/have a fever?

   Go to clinic/hospital ( ) Go to Pharmacy ( )

   Home treatment/self-medication ( ) Do nothing ( )

   Others____

3. What determines/influences your action to seek care if your child(ren) has a fever?

   Condition of the child ( ) Cost involved ( )

   Time availability ( ) Others___________________________________

4. What do you do when an antimalarial medication is prescribed for your sick child(ren)?

   Administer the full treatment course ( )

   Stop administration as soon as child begins to show improvement ( )

   Administer to other siblings who are sick or not sick with malaria ( )

   Others____

5. How long do wait before seeking medical help if your child has a fever?

   Within 24h ( ) 2-5 days ( )

   More than 7 days ( ) Not sure ( ) Others______________________
