# Supplementary material for: A Cross-Sectional Survey on the Malaria Control and Prevention Knowledge, Attitudes, and Practices of Caregivers of Children Under-5 in the Western Area of Sierra Leone
Source: Trop Med Infect Dis. 2022 Jun 28;7(7):120. doi: 10.3390/tropicalmed7070120 (PMC9319430; doi:10.3390/tropicalmed7070120)
Supplement: Supplementary file 1 [file tropicalmed-07-00120-s001.zip › Supplementary file S2.pdf]

Scoring system to evaluate the levels of Knowledge, Attitude and Practice (KAPs) among the caregivers of children under-5. The scoring criteria are as follows:

- Knowledge (9 point):

1. Know that malaria is caused by parasites through mosquito bites (1 point);
2. Mention the breeding places of mosquitoes, stagnant water(1 point); one of other places that suitable for mosquitoes to rest including bushes/dirty places, dark places/sheds or other fit places (1 point);
3. Mention the symptom, fever (1 point); headache (1 point); three or more other common symptoms including body and joint pains, vomiting, loss of appetite, and other fit symptom (1 point);
4. Malaria can be prevented (1 point);
5. Malaria can be cured (1 point);
6. Malaria can lead to death (1 point).

- Attitude (6 point):

1. Awareness of the seriousness of malaria (1 point);
2. Awareness of sleeping under a bed net (1 point); thinking the bed net is treated (1 point) and would always use the bed net (1 point);
3. Thinking that mosquito bites at the night time most (1 point);
4. Thinking that Artemisinin-based Combination Therapies (ACTs) is the best treatment for malaria (1 point).

- Practice (9 point):

1. Take the measure, sleeping under insecticide-treated bed net (2 point), choose one (1 point) or more(2 point) measures including: insecticide spray, wearing protective clothing, and mosquito repellant; two or more measures including clearing bushes around the house, getting rid of stagnant water, regular clean-ups around the house and other fit measures (1 point);
2. Go to clinic/hospital (1 point) within 24h (1 point) when child has fever;
3. Seeking care according the condition of child (1 point);
4. Administer the full treatment course for sick child (1 point).

The level of KAPs are recorded to binary level. High/low knowledge, positive/negative attitude and good/bad practice levels were defined as scores above and below the mean score, respectively.
