# Supplementary material for: A Cross-Sectional Survey on the Malaria Control and Prevention Knowledge, Attitudes, and Practices of Caregivers of Children Under-5 in the Western Area of Sierra Leone
Source: Trop Med Infect Dis. 2022 Jun 28;7(7):120. doi: 10.3390/tropicalmed7070120 (PMC9319430; doi:10.3390/tropicalmed7070120)
Supplement: Supplementary file 1 [file tropicalmed-07-00120-s001.zip › Supplementary file S3.pdf]

Table S1 Comparison of knowledge towards malaria between caregivers living in rural and urban districts

Variables	Total (n, %)	Districts (n, %)		*P*
		Urban(173)	Rural(177)	
**Ever heard of malaria?**				**0.620[b]**
Yes	347(99.1)	171(98.8)	176(99.4)	
No	3(0.9)	2(1.2)	1(0.6)	
**Source(s) of information about malaria[Ψ] (Response rate: 99.1%)**				**0.858[a]**
Television	61(17.4)	35(20.2)	26(14.7)	**0.172**
Internet/social media	5(1.4)	3(1.7)	2(1.1)	**0.682**
Radio	211(60.3)	106(61.3)	105(59.3)	**0.634**
Health workers/facilities	309(88.3)	149(86.1)	160(90.4)	**0.214**
Printed materials (billboards/handbills, etc)	28(8.0)	14(8.1)	14(7.9)	**0.950**
Others	93(26.6)	46(26.6)	47(26.6)	**0.994**
**What causes malaria?**				**0.592[a]**
Parasite through mosquito bite	317(90.6)	157(90.8)	160(90.4)	
Eating too much	1(0.3)	1(0.6)	0(0.0)	
Close contact with a person who has malaria	1(0.3)	0(0.0)	1(0.6)	
Don't know	17(4.9)	7(4.0)	10(5.6)	
Others	14(4.0)	8(4.6)	6(3.4)	
**Resting/breeding places of mosquitoes[Ψ]**				**0.332[a]**
Stagnant water	179(51.1)	84(48.6)	95(53.7)	**0.338**
Bushes/dirty places	303(86.6)	152(87.9)	159(89.8)	**0.484**
Dark places/sheds	41(11.7)	21(12.1)	20(11.3)	**0.807**
Don't know	28(8.0)	11(6.4)	17(9.6)	**0.263**
Others	9(2.6)	7(4.0)	2(1.1)	**0.102**
**Signs & symptoms of malaria[Ψ]**				**0.094[a]**
Fever	251(71.7)	120(69.4)	131(74.0)	**0.334**
Headache	32(9.1)	12(6.9)	20(11.3)	**0.157**
Body & joint pains	94(26.9)	40(23.1)	54(30.5)	**0.119**
Vomiting	150(42.9)	82(47.4)	68(38.4)	**0.09**
Loss of appetite	108(30.9)	50(28.9)	58(32.8)	**0.434**
Don’t know	9(2.6)	2(1.2)	7(4.0)	**0.174**
Others	205(58.6)	112(64.7)	93(52.5)	**0.020***
**Can malaria be prevented?**				**0.077[a]**
Yes	287(82.0)	150(86.7)	137(77.4)	
No	8(2.3)	3(1.7)	5(2.8)	
Don't know	55(15.7)	20(11.6)	35(19.8)	
**Can malaria be cured?**				**0.050[b]**
Yes	338(96.6)	171(98.8)	167(94.4)	
No	4(1.1)	0(0.0)	4(2.3)	
Don't know	8(2.3)	2(1.2)	6(3.4)	
**Can malaria lead to death?**				**0.947[a]**
Yes	319(91.1)	157(90.8)	162(91.5)	
No	13(3.7)	7(4.0)	6(3.4)	
Don't know	18(5.1)	9(5.2)	9(5.1)	

[Ψ] Multiple responses; [a] Pearson Chi-square; [b] Fisher’s Exact Test; [*] *P*<0.05; [**] *P*<0.01; [***] *P*<0.001
