# Supplementary material for: A Cross-Sectional Survey on the Malaria Control and Prevention Knowledge, Attitudes, and Practices of Caregivers of Children Under-5 in the Western Area of Sierra Leone
Source: Trop Med Infect Dis. 2022 Jun 28;7(7):120. doi: 10.3390/tropicalmed7070120 (PMC9319430; doi:10.3390/tropicalmed7070120)
Supplement: Supplementary file 1 [file tropicalmed-07-00120-s001.zip › Supplementary file S4.pdf]

Table S2 Comparison of attitudes towards malaria between caregivers living in rural and urban districts

Variables	Total	Districts (n, %)		*P*
	(n, %)	Urban(173)	Rural(177)	
**Do you think malaria is a very serious health problem?**				**0.500**[a]
Yes	315(90.0)	160 (92.5)	155(87.6)	
No	11(3.1)	4 (2.3)	7(4.0)	
Not sure	19(5.4)	7 (4.0)	12(6.8)	
Don't know	5(1.4)	2 (1.2)	3(1.7)	
**Do you and your child(ren) sleep under a bed net?**				**0.000**[a***]
Yes	272(77.7)	121 (69.9)	151(85.3)	
No	78(22.3)	52 (30.1)	26(14.7)	
**If Yes (Response rate: 77.7%), is the bed net:**				**0.883**[b]
Treated	213(78.3)	94 (77.7)	119(78.8)	
Not treated	59(21.7)	27 (22.3)	32(21.2)	
**How often do you and your child(ren) use the bed net?**				**0.204**[b]
Always	233(85.7)	100 (82.6)	133(88.1)	
Sometimes	39(14.3)	21 (17.4)	18(11.9)	
**If No (n = 78, 22.3%), why?**				**0.130**[a]
I can't afford it	36(46.2)	23 (44.2)	13(50.0)	
It is not readily available	25(32.1)	19 (36.5)	6(23.1)	
My child(ren) and I don't like sleeping under a bed net	5(6.4)	5 (9.6)	0 (0.0)	
I don't think it is important	3(3.8)	1 (1.9)	2(7.7)	
Others	9(11.5)	4 (7.7)	5(19.2)	
**At what time of day do you think mosquitoes bite most?**				**0.908**[a]
Daytime	5(1.4)	2 (1.2)	3(1.7)	
Night time	308(88.0)	151 (87.3)	157(88.7)	
Anytime	30(8.6)	16 (9.2)	14(7.9)	
Don't know	7(2.0)	4 (2.3)	3(1.7)	
**What do you think is the best treatment for malaria?**				**0.245**[a]
ACTs	228(65.1)	116 (67.1)	112 (63.3)	
Paracetamol	2(0.6)	2 (1.2)	0 (0.0)	
Traditional/herbal medications	3(0.9)	1 (0.6)	2 (1.1)	
Don't know	112(32.0)	50 (28.9)	62 (35.0)	
Others	5(1.4)	4 (2.3)	1 (0.6)	

[a] Pearson Chi-square; [b] Fisher's Exact Test; [*] P<0.05; [**] P<0.01; [***] P<0.001;

ACTs：Artemisinin-based Combination Therapies
