# Supplementary material for: A Cross-Sectional Survey on the Malaria Control and Prevention Knowledge, Attitudes, and Practices of Caregivers of Children Under-5 in the Western Area of Sierra Leone
Source: Trop Med Infect Dis. 2022 Jun 28;7(7):120. doi: 10.3390/tropicalmed7070120 (PMC9319430; doi:10.3390/tropicalmed7070120)
Supplement: Supplementary file 1 [file tropicalmed-07-00120-s001.zip › Supplementary file S5.pdf]

Table S3 Comparison of practices towards malaria between caregivers living in rural and urban districts

Variables	Total (n, %)	Districts (n, %)		*P*
		Urban(173)	Rural(177)	
**Protective measures against mosquito bites**$^{\Psi}$				
Sleeping under an ITN	214(61.1)	90(52.0)	124(70.1)	**0.001*****
Mosquito repellant	143(40.9)	76(43.9)	67(37.9)	**0.248**
Regular clean-ups around the house	111(31.7)	51(29.5)	60(34.0)	**0.374**
Wearing protective clothing	88(25.1)	46(26.6)	42(23.7)	**0.537**
Insecticide spray	87(24.9)	45(26.0)	42(23.7)	**0.621**
Getting rid of stagnant water	29(8.3)	12(6.9)	17(10.0)	**0.365**
Clearing bushes around the house	12(3.4)	11(6.4)	1(0.6)	**0.003****
Others	21(6.0)	8(4.6)	13(7.3)	**0.284**
**What is the first thing you do when your child(ren) has fever?**				**0.640**^{a}
Go to clinic/hospital	270(77.1)	130(75.1)	140(79.1)	
Go to Pharmacy	12(3.4)	7(4.0)	5(2.8)	
Home treatment/self-medication	68(19.4)	36(20.8)	32(18.1)	
**What determines/influences your action to seek care if your child(ren) has fever?**				**0.838**^{a}
Condition of the child	327(93.4)	160(92.5)	167(94.4)	
Cost involved	13(3.7)	7(4.0)	6(3.4)	
Time availability	6(1.7)	4(2.3)	2(1.1)	
Others	4(1.1)	2(1.2)	2(1.1)	
**What do you do when an antimalarial medication is prescribed for your sick child(ren)?**				**0.000**^{a***}
Administer the full treatment course	250(71.4)	138(79.8)	112(63.3)	
Stop administration as soon as child begins to show improvement	45(12.9)	22(12.7)	23(13.0)	
Administer to other siblings who are sick/not sick with malaria	7(2.0)	4(2.3)	3(1.7)	
Others	48(13.7)	9(5.2)	39(22.0)	
**How long do you wait before seeking medical help, if your child has fever?**				**0.130**^{a}
Within 24h	191(54.6)	86(49.7)	105(59.3)	
2-5 days	158(45.1)	86(49.7)	72(40.7)	
Others	1(0.3)	1(0.6)	0(0.0)	

$^{\Psi}$ Multiple responses; ^{a} Pearson Chi-square; ^{b} Fisher's Exact Test; * p<0.05; ** p<0.01; *** p<0.001

ITN：Insecticides Treated Nets
