# Supplementary material for: A Cross-Sectional Survey on the Malaria Control and Prevention Knowledge, Attitudes, and Practices of Caregivers of Children Under-5 in the Western Area of Sierra Leone
Source: Trop Med Infect Dis. 2022 Jun 28;7(7):120. doi: 10.3390/tropicalmed7070120 (PMC9319430; doi:10.3390/tropicalmed7070120)
Supplement: Supplementary file 1 [file tropicalmed-07-00120-s001.zip › Supplementary file S6.pdf]

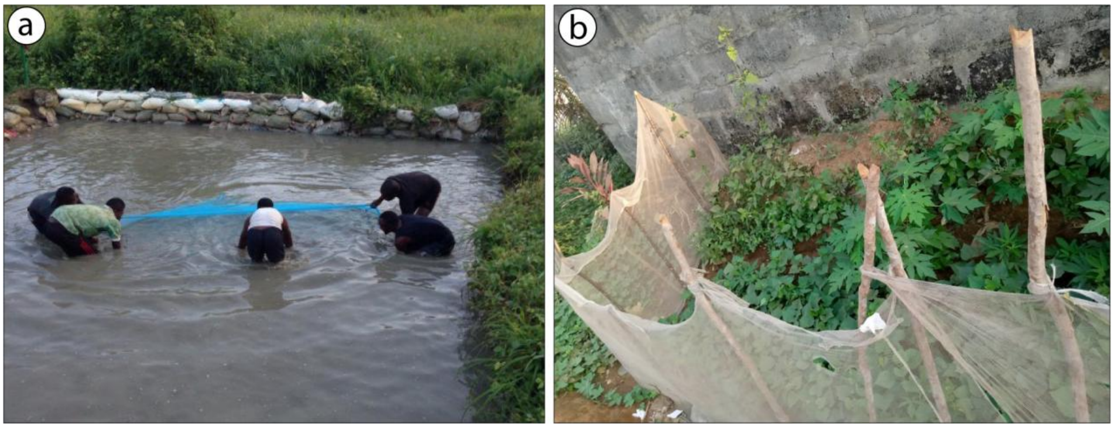

Figure S1 Images of mosquito bed net misuse in Sierra Leone. **(a)**: Some individuals were doing pond fishing using mosquito bed nets. **(b)**: Small backyard gardens were covered with mosquito bed nets. Misuse of the bed nets like these is common especially in rural and provincial areas of Sierra Leone.
