# Supplementary material for: A Cross-Sectional Survey on the Malaria Control and Prevention Knowledge, Attitudes, and Practices of Caregivers of Children Under-5 in the Western Area of Sierra Leone
Source: Trop Med Infect Dis. 2022 Jun 28;7(7):120. doi: 10.3390/tropicalmed7070120 (PMC9319430; doi:10.3390/tropicalmed7070120)
Supplement: Supplementary file 1 [file tropicalmed-07-00120-s001.zip › Supplementary file S7.pdf]

Table S4 KAPs levels of caregivers towards malaria control/prevention based on the scoring system

Items	Variables	Knowledge		Attitudes		Practices	
		High	Low	Positive	Negative	Good	Bad
Age (years)	15-20	42	26	54	14	30	38
	21-25	87	41	77	51	53	75
	26-30	70	25	76	19	42	53
	> 30	49	10	45	14	26	33
Gender	Male	10	0	5	5	1	9
	Female	238	102	247	93	150	190
Religion	Christian	90	21	87	24	42	69
	Muslim	158	81	165	74	109	130
Relationship	Mother	225	99	238	86	145	179
	Father	10	0	5	5	1	9
	Others1	13	3	9	7	5	11
Children under-5	= 1	154	64	156	62	86	132
	≥ 2	94	38	96	36	65	67
Other children above-5	= 0	116	43	113	46	66	93
	≥ 1	132	59	139	52	85	106
District	Urban	120	53	114	59	70	103
	Rural	128	49	138	39	81	96
Marital status	Single	52	16	49	19	32	36
	Married	170	75	179	66	107	138
	Consensual union	26	11	24	13	12	25
Education	None	58	37	61	34	47	48
	Primary2	19	11	24	6	12	18
	Secondary3	149	49	143	55	81	117
	University	22	5	24	3	11	16
Occupational status	Unemployed	105	57	118	44	72	90
	Employed	115	40	112	43	74	81
	Student	28	5	22	11	5	28
